# Vpr content of HIV-1 virions determines infection of resting peripheral blood CD4^+^ lymphocytes

**DOI:** 10.1186/1742-4690-10-S1-P92

**Published:** 2013-09-19

**Authors:** Anouk Van Nuffel, Francis Impens, Ann Baeyens, Stijn Vanhee, Wojciech Witkowski, Elien Vandermarliere, Hanne Vanderstraeten, Evelien Naessens, Kathleen Vanlandeghem, Sophie Vermaut, Kathleen Moens, Petra Van Damme, Kris Gevaert, Bruno Verhasselt

**Affiliations:** 1 Department of Clinical Chemistry, Microbiology and Immunology, Ghent University, Ghent, Belgium; 2 Department of Biochemistry, Ghent University and VIB Department of Medical Protein Research, Ghent University, Ghent, Belgium

## Background

The HIV-1 Vpr protein is a 14 kDa accessory protein, required for efficient replication in macrophages. Vpr is incorporated into HIV virions, believed to participate in the docking of the HIV-1 pre-integration complex to the nucleus and to facilitate it’s transport through the nuclear pore. By inducing G2 arrest, Vpr favors transcription from the HIV-1 LTR, which it also transactivates.

We noticed two N-terminal amino acids of the HIV-1, SIVmac and SIVcpz Vpr proteins are fully conserved. This N-terminal motif is predicted to be a NatB substrate motif (i.e., Met-Glu-), expected to lead to full Nt-acetylation of the protein, but it also allows the conservation of the Kozak consensus sequence (A/GCCAUGG), critical for efficient protein translation. Nt-acetylation is one of the most common protein modifications in eukaryotes and is believed to affect protein stability, degradation and function.

## Methods

Nt-acetylation of Vpr was detected by COFRADIC (Combined FRActional Diagonal Chromatography) in HIV-1 infected Jurkat cells, and confirmed with selected reaction monitoring mass spectrometry on transfected cells. Point mutations were introduced in the Vpr protein, abolishing Nt-acetylation in part or completely, and modifying the Kozak consensus sequence. Mutants were cloned into a retroviral vector. Protein expression, translation, degradation, localization, and function (G2 arrest, apoptosis and transactivation) was evaluated in Jurkat and/or 293T cells. In parallel, the Vpr protein of a NL4.3 HIV-1 reporter virus was mutated similarly. Vpr WT and mutant protein expression, incorporation into viral particles and viral infectivity was evaluated in resting and activated primary CD4+ T cells.

## Results

We showed full Nt-acetylation of Vpr by COFRADIC in infected Jurkat cells and set out to determine whether Vpr’s amino-terminal sequence is conserved for functional Nt-acetylation, or to allow optimal protein translation.

Results show Vpr protein expression levels to be reduced by mutations affecting Nt-acetylation. However, when protein translation was inhibited, mutants versus wild-type Vpr were not degraded faster, suggesting Nt-acetylation does not protect the protein from degradation. The reduced steady state levels could be the result of mutated Kozak consensus sequence on translation efficiency, still under investigation. When overexpressed, mutants retained Vpr functions tested, also suggesting Nt-acetylation is not necessary for Vpr function *per se.*

However, in the context of a replication competent HIV-1 virus, the reduced mutant Vpr levels in infected cells resulted in diminished incorporation into HIV particles. In resting but not in activated T cells, infection rates with Vpr N-terminus mutants were reduced down to levels of Vpr deleted HIV-1.

## Conclusions

Altogether, our results show efficient infection of resting CD4^+^ lymphocytes to be dose-dependent on Vpr expression levels. N-terminus conservation is important for sufficient Vpr protein levels in infected cells and virions, but Nt-acetylation does not affect protein function *per se*, nor does it influence protein half-life.

